# Organ-preserving embolization of a giant splenic hemangioma in an adult

**DOI:** 10.1186/s42155-024-00491-1

**Published:** 2024-11-08

**Authors:** Manos Siderakis, Stamatia Dodoura, George Gkeneralis, Viktoria Kartsouni, Myrsini Gkeli

**Affiliations:** Interventional Unit of Radiology, Agios Savas Anticancer Hospital, Alexandras Avenue 171, Athens, 11522 Greece

**Keywords:** Spleen, Hemangioma, Organ-preserving, Splenic artery embolization

## Abstract

Splenic hemangioma is a rare benign tumor, often found incidentally. Large hemangiomas bear a high risk of spontaneous rupture, an acute condition often life-threatening that requires immediate intervention. The spleen’s vital role in immunity and blood filtration mandates organ-preserving management of conditions such as hypersplenism or large lesions such as hemangiomas. A 51-year-old patient was referred to our department for abdominal pain due to a giant spleen hemangioma (6,6 × 6,3 × 5,6) cm in size. The patient was treated with hemangioma embolization through the splenic artery branches that fed the lesion, without any complications. Two years post-embolization the patient mentioned no symptoms and the hemangioma was measured (3 × 2) cm.

**Background** The patient was referred to our department due to the large size of the lesion and the patient’s preference to avoid surgery and maintain the spleen. Our case report aims to underline (a) the safety and feasibility of the protocol among interventional radiologists, surgeons, and patients, (b) the benefit of relieving symptoms without losing a vital organ, and (c) the lower complication rate and hospital days compared with surgery. There are few reported cases of embolization of large spleen hemangioma. While Choi et al. (BMC Pediatr. 2018 12;18(1):354) report a similar case in a child. This case report describes the procedure in a stable adult and provides a 2-year follow-up.

**Graphical Abstract:**

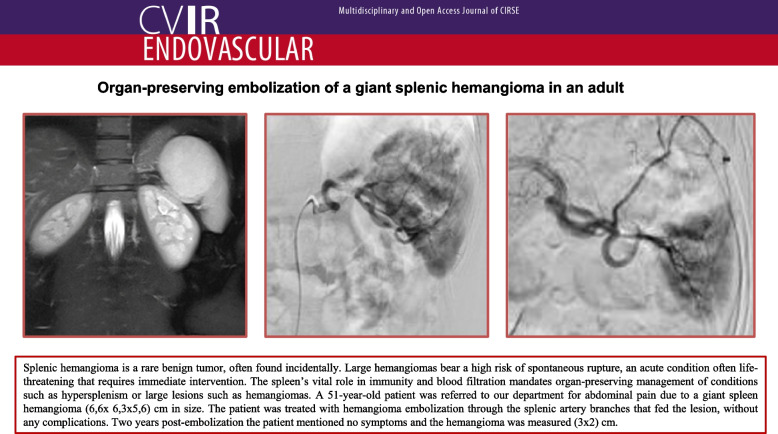

## Introduction

The spleen is the largest lymphoid organ, providing a proper environment for lymphocytes to proliferate and for aging or abnormal red blood cells to be removed. Since it does not play a pivotal role in maintaining vital functions, clinicians often neglect it. However, spleen compromise or surgical removal due to disease or trauma can affect immunity, such as in the case of the post-operative Overwhelming Post-Splenectomy Infection (OPSI). OPSI, a rapidly progressive and potentially fatal infection within 24 h, has an incidence of 4,4% and a mortality rate of 50–80% [1].

Hemangiomas, often found incidentally, are the second most common focal lesions of the spleen after simple cysts and are found in 0,03%-14% of autopsy series [2]. They originate from the sinusoidal epithelium and comprise non-encapsulated vascular channels [3]. Mostly asymptomatic, splenic hemangiomas can be solitary or multiple. The main complications are rupture, hypersplenism, and rare malignant transformation [4]. On ultrasound, splenic hemangiomas are well-defined, round, and of variable echogenicity, usually hyperechoic. On contrast-enhanced CT, they typically show early, peripheral, nodular enhancement gradually progressing to a homogenous pattern [4]. However, enhancement can also be early homogenous or early peripheral with centripetal progression. On MRI, splenic hemangiomas show T2-weighted hyperintensity. Imaging of splenic hemangiomas may be affected by a cystic component, calcifications, fibrosis, and hemorrhage.

Splenic embolization using autologous blood clot was first introduced in 1973 by Maddison to treat hypersplenism [5]. In 1979, Spigos et al. treated 13 patients with hypersplenism via transcatheter partial splenic embolization (PSE), providing a protocol of splenic artery embolization, antibiotic prophylaxis, and adequate pain control [6]. PSE leads to ischemia of the whole organ or devascularization of a specific splenic lesion or area when applied to selected arteries. PSE is associated with lower morbidity and mortality than splenectomy, overcoming the disadvantages of a splenectomy such as high blood loss, longer hospital stay, and OPSI [7, 8]. On the other hand, PSE may be followed by a postembolization syndrome in up to 30% of cases, which includes fever, abdominal pain, nausea, and vomiting [4]. Other PSE complications include pneumonia, non-traumatic rupture, splenic abscess, peritonitis, and contrast-mediated nephropathy.

## Case report

**Fig. 1:**
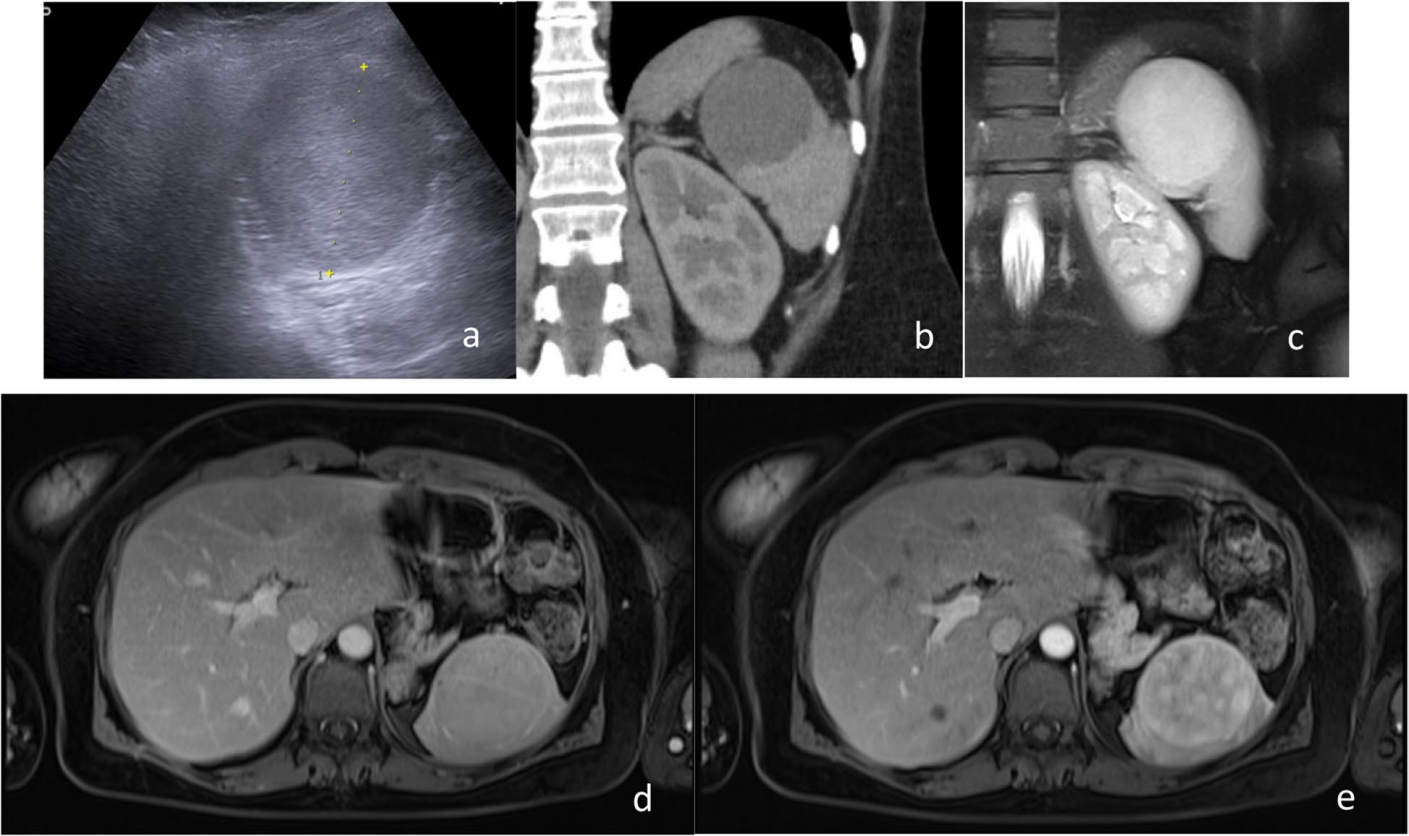
**a** A well-defined, round, mostly hyperechoic lesion is noted on the upper lobe of the spleen via ultrasound. **b** The lesion shows early, peripheral, nodular enhancement on CT and is measured (6,6 × 6,3 × 5,6) cm. **c** the splenic hemangioma shows T2-weighted hyperintensity on MRI. **d** The mass shows early peripheral enhancement and **e** late central enhancement on MRI T1-weighted post-gadolinium images

A 51-year-old female patient with no medical history of trauma or abdominal surgery, was referred to our department to evaluate recent clinical and imaging findings. She complained of persistent left upper quadrant abdominal discomfort during the last months. On ultrasound, a round, well-defined, mostly hyperechoic lesion was identified on the upper pole of the spleen (Fig. 1a). On contrast-enhanced CT the lesion was measured (6,6 × 6,3 × 5,6) cm (maximal axial x coronal x sagittal dimension) and showed early, peripheral, and nodular enhancement (Fig. 1b, c). On MRI, the lesion showed T2 hyperintensity and initially peripheral and gradually homogenous gadolinium enhancement (Fig. 1d, e). The lesion occupying more than half of the patient’s spleen was mostly compatible with hemangioma. The patient was informed of the advantages and risks of embolization and consented to proceed.

**Fig. 2:**
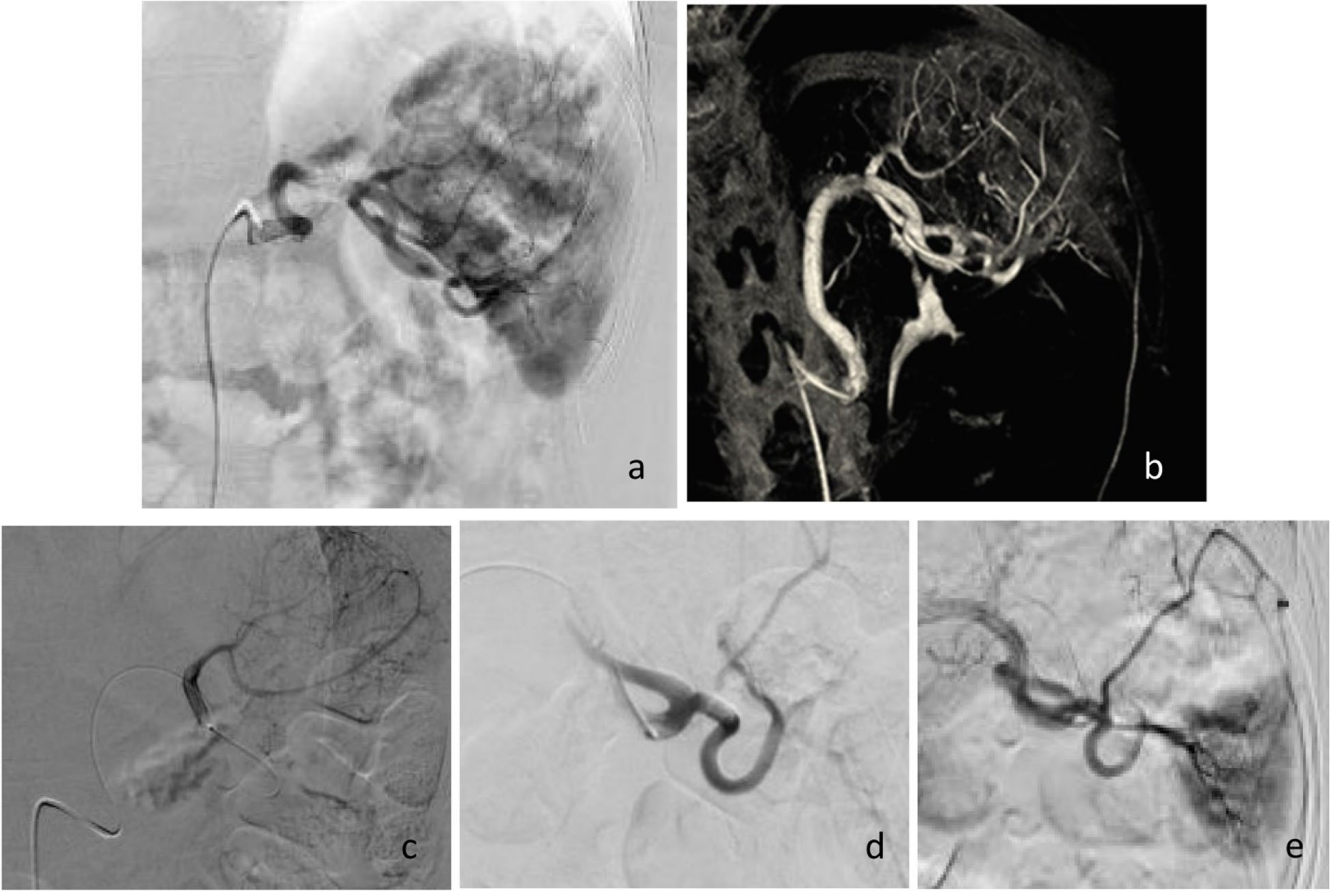
**a** DSA of the splenic artery and its branching pattern within the hemangioma. **b** CBCT provides 3D imaging of the hemangioma, its upper and lower feeding artery network, and its relation to close anatomical structures. Selective catheterization of splenic artery branches that feed the upper (**c**) and lower **d** half of the hemangioma. **e** Post-embolization image depicting the absence of blood supply to the hemangioma without affecting the remaining central and lower pole spleen parenchyma

The patient was positioned supine and received femoral artery puncture using the Seldinger technique under local anesthesia. A 5F arterial sheath was placed (Merit Medical Systems, Inc, South Jordan, USA), and a 5F catheter (Cobra I, Cordis Corporation, Florida, USA) was then used to perform angiography of the celiac trunk, splenic artery, and branching network (Fig. 2a). Cone-beam CT (CBCT) assisted in depicting the 3-dimensional architecture of the vascular tree and allowing selective targeting of the lesion’s feeding vessels (Fig. 2b). A 2,6F microcatheter (Asahi Intecc Co., LTD, Japan) was inserted into the splenic artery and hyper-selective angiography of the hemangioma feeding vessels was performed (Fig. 2c, d). Embolization started with Embosphere microspheres (BioSphere Medical, S.A. Paris Nord 2, France) of size 100-300 μm for distal embolization and gradually increasing range for proximal embolization; 300-500 μm, 500-700 μm, and 700-900 μm. Following embolization, angiography demonstrated devascularization of the hemangioma and normal blood supply of the remaining spleen parenchyma (Fig. 2e). During the procedure, the patient was provided with analgesics and was continuously monitored. No complications were noted during or after the procedure. The patient was discharged 24 h post-embolization. No vaccination was performed before the procedure since the patient was immunocompetent and would retain more than 30% of spleen volume [6]. Prophylactic antibiotic (1 g cefuroxime/day for 5 days) was prescribed post-procedure.

**Fig. 3:**
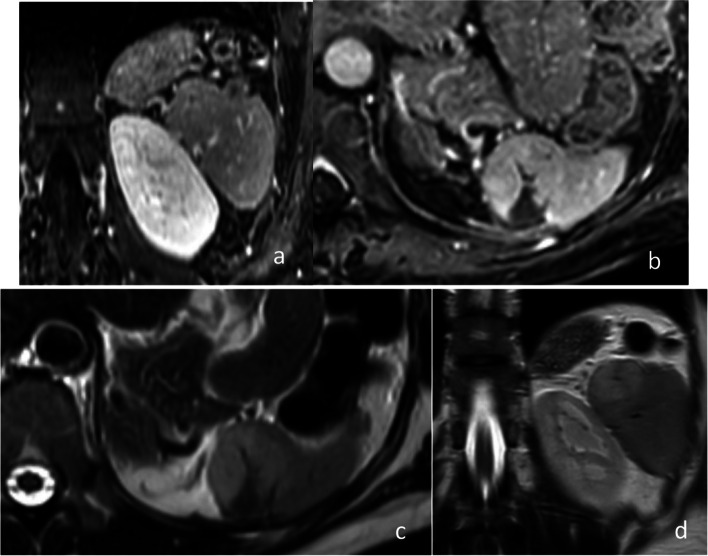
MRI examination 2 years post-embolization. **a** Normal spleen parenchyma (Coronal T1-Gd-weighted). **b** The known hemangioma on the upper spleen pole is now measured (3 × 2) cm (Axial T1-Gd-weighted). The lesion appears with moderate signal on axial (**c**) and coronal (**d**) T2-weighted images. A triangular area of post-embolization infarction is recognized (**b**, **c**)

On a 2-year post-embolization follow-up MRI exam, normal parenchyma of the spleen showed an impressive “decompression” appearance (Fig. 3a). A lesion of (3 × 2) cm was noted on the upper lobe of the spleen with moderately high T2-weighted signal and mild segmental, peripheral enhancement on contrast-enhanced T1-weighted images (Fig. 3b-d). The patient reported no symptoms.

## Discussion

Large hemangiomas have a higher risk of spontaneous rupture and life-threatening hemorrhage [2]. Therefore, hemangiomas > 4 cm are recommended to be treated [2, 7]. Most pathologies of the spleen have been traditionally managed with surgical removal of the organ. However, its well-established role in blood filtration, immunity, and hemopoiesis has favored organ-preserving therapies when applicable. Additionally, splenectomy can be followed by OPSI, a potentially fatal complication. Indications for splenic embolization include abdominal trauma, hypersplenism, and neoplasm [7]. The technique is contra-indicated in secondary splenomegaly and hypersplenism due to terminal stage original disease and severe infection that may predispose to spleen abscess formation after the procedure [9]. In the case of a sizeable spleen hemangioma, selective embolization of its feeding vessels can induce local ischemia and infarction leading to lesion shrinkage, without affecting the remaining normal parenchyma.

Embolic material for partial splenic embolization includes gelatin sponge (Gelfoam), metallic coils, n-butyl cyanoacrylate (NBCA), polyvinyl alcohol (PVA) particles, acrylic polymer microspheres (embospheres), PVA hydrogel beads with an acrylic polymer, hydropearl microspheres, and hydrogel microspheres coated with Polyzene-F [9, 11]. Compared with gelatin sponge, PVA particles and embospheres are permanent embolic materials, smaller and available in specific size ranges. Embospheres are spherical, whereas PVA particles have sharp margins. In a meta-analysis of 10 studies between 1991 and 2013, Rong et al. concluded that the embolic material does not affect the primary success rate of splenic artery embolization [12]. Coils and microspheres show a lower rate of adverse clinical outcomes when compared with gelfoam [12, 13]. In a retrospective study of 179 patients in whom partial splenic embolization for hypersplenism was performed, three commonly used embolic agents were compared: gelatin sponge, embospheres, and PVA particles [11]. Permanent embolic agents (embospheres and PVA particles) were associated with a lower incidence of major complications and fewer hospital days but caused more severe and longer post-procedural pain [11]. N-butyl cyanoacrylate (NBCA) is considered a rapid embolizing agent, suitable for long sections, even in cases of affected coagulation state [14]. However, the embolization area cannot be reliably pre-assessed due to the glue’s unpredictable behavior during injection [14].

Embolization of the spleen is a safe, less invasive option than splenectomy with reduced risk of bleeding. A postembolization syndrome of fever and abdominal pain is typically unavoided and self-limited. In a randomized prospective study of 40 patients with hypersplenism divided into two equal groups, PSE was compared with traditional splenectomy [15]. Postoperative complications were comparable in the two groups, but pain, operation time, and hospital days were reduced in the PSE group. Moreover, PSE does not require blood transfusion and can be performed under local anesthesia.

Our case study demonstrates a gradual shrinkage of a spleen hemangioma, occupying more than half of the organ, within two years post-embolization (images not shown). Although no obvious spleen dysfunction was verified before embolization, a functional and histopathological study of a highly compressed spleen due to a giant hemangioma would be particularly interesting. It is worth mentioning that embolization of a spleen hemangioma has also been implemented before splenectomy, both in stable, non-rupture conditions [8] and in the case of life-threatening spontaneous hemangioma rupture [10].

## Conclusion

A symptomatic giant splenic hemangioma was treated with selective embolization of its feeding splenic artery branches. The patient avoided losing a vital organ for immunity and blood filtration if the alternative of splenectomy had been preferred. Moreover, the procedure’s safety and feasibility should highlight the role interventional radiologists may play in similar cases. Finally, the low complication rate of splenic hemangioma embolization ensures fewer hospital days and lower healthcare costs.

## Data Availability

All data generated or analyzed during this study are included in this published article.
